# Seasonal Dynamics and Factors Shaping Aquatic Insect Assemblages in Mountain Streams of the Pannonian Lowland Ecoregion

**DOI:** 10.3390/insects16040344

**Published:** 2025-03-26

**Authors:** Viktorija Ergović, Dubravka Čerba, Bojana Tubić, Boris Novaković, Miran Koh, Zlatko Mihaljević

**Affiliations:** 1 Department of Biology, University of Osijek, Cara Hadrijana 8/a, 31000 Osijek, Croatia; viktorija.ergovic@biologija.unios.hr (V.E.); miran.koh@biologija.unios.hr (M.K.); 2 Department of Assessment and Aquatic Ecosystems Research, National Water Reference Laboratory of Slovakia, Water Research Institute, Nábr. arm. gen. L. Svobodu 5 (7), 81249 Bratislava, Slovakia; 3 Department for Hydroecology and Water Protection, Institute for Biological Research “Siniša Stanković”, National Institute of the Republic of Serbia, University of Belgrade, Bulevar despota Stefana 142, 11108 Belgrade, Serbia; bojana@ibiss.bg.ac.rs; 4 National Laboratory, Serbian Environmental Protection Agency, Ministry of Environmental Protection, Žabljačka 10a, 11160 Belgrade, Serbia; boris.novakovic@sepa.gov.rs; 5 Department of Biology, Faculty of Science, University of Zagreb, Horvatovac 102a, 10000 Zagreb, Croatia; zlatko.mihaljevic@biol.pmf.unizg.hr

**Keywords:** community structure, stream macrozoobenthos, functional feeding groups, EPT, Coleoptera, Odonata, Croatia

## Abstract

This study focused on the aquatic insect orders that serve as biological indicators. Ephemeroptera, Plecoptera, Trichoptera, Coleoptera, and Odonata were studied with their relationships to physical, chemical, hydrological and morphological variables in 14 streams on three Croatian mountains: Papuk, Medvednica and Psunj. From 675 samples collected in three dominant sediment microhabitats (e.g., macrolithal and mesolithal) in each stream, we identified 130 macroinvertebrate taxa. The most abundant group was Ephemeroptera (36 taxa). Aquatic insect assemblages between Papuk and Psunj showed higher similarity due to the geographical proximity of these two mountains, which is expressed as spatial connectivity. In spring, greater variability and diversity of investigated aquatic insects were observed due to the phenology of studied insects. The combination of environmental and morphological properties (e.g., oxygen concentration and substrate type) had the most significant influence on shaping the assemblage of aquatic insects of Psunj, while morphological properties were the most important in shaping aquatic insect assemblages on Papuk (e.g., streambed width) and Medvednica (e.g., elevation). *Baetis* sp. was the dominant taxon in summer, *Protonemura montana* in spring, and *Leuctra* sp. in autumn. Seasonal shifts in environmental conditions lead to functionally unique communities that utilize the pool of available seasonal resources. The dominance of feeding groups varied by season, with scrapers and collector–gatherers being most prevalent in summer, while shredders were dominant in spring. The results on the relationships between environmental characteristics and species richness, functional structure, and distribution of aquatic insects in mountain streams provide important clues for future research and conservation strategies in these vulnerable ecosystems.

## Simple Summary

Mountain streams are remarkable ecosystems characterized by particular ecological features that are highly sensitive to changes in temperature and water quality. Protection of such ecosystems and studies of their biodiversity is of great importance. This research on aquatic insect communities was conducted across three Croatian mountains: Papuk, Medvednica, and Psunj, located in the Pannonian Lowland Ecoregion, in 14 mountain streams. The ecoregion is an area with similar species assemblages and environmental characteristics for a specific geographical region. It focused on mayflies, stoneflies, caddisflies, beetles, and dragonflies, which are excellent bioindicators of water quality. In 675 samples, 130 insect taxa (with 60 species) were identified. Seasonal variations were tested and revealed unique patterns: different species dominated, like *Baetis* sp. in summer, *Protonemura montana* in spring, and *Leuctra* sp. in autumn. Streams on Papuk and Psunj showed greater ecological similarities, likely due to their proximity, and water quality, together with stream morphology, which strongly influenced insect communities. In addition to high biodiversity, two endangered species were identified: dragonfly *Cordulegaster heros* and stonefly *Taeniopteryx hubaulti*. Our results emphasize the importance of understanding how environmental factors affect aquatic insect taxa richness and distribution in sensitive lotic mountain ecosystems.

## 1. Introduction

Mountain streams as a complex type of ecosystem can be defined by their hydrological, morphological, and environmental properties, including the slope gradient, low water temperature, high flow velocity, characteristic hydrology, and substrate composition [1,2]. The definition of a mountain stream is not only based on elevation. There are several factors that differentiate between lowland and mountain streams, and stream classification varies according to region, climate, and local topography [3]. They are usually surrounded by forests and characterized by consistently cold water and diverse types of substrate, mainly boulders and cobbles, while gravel and fine sediment are generally absent, but dominant in lowland streams [4,5,6]. Available food sources are mainly from the leaf litter of the surrounding vegetation and there is less of it than in the lowland rivers [7]. These conditions create diverse microhabitats and attract a variety of aquatic life, representing a hotspot for macroinvertebrate fauna [8,9]. The heterogeneity of the habitat is primarily related to the altitude. Altitudinal factors (i.e., environmental gradients, spatial connectivity, ecological interactions, and landscape geomorphology) have jointly affected biological diversity in streams, with emphasis on differences in abundance and community structure of aquatic organisms [2,10].

Aquatic insects play a crucial role in the ecosystem dynamics of mountain streams and serve as important indicators of water quality and environmental health [11,12,13]. Aquatic insect assemblages in streams are distributed longitudinally, mainly based on water temperature and channel stability [14,15]. Insect fauna in mountain streams consists primarily of Ephemeroptera, Plecoptera, and Trichoptera (EPT). The EPT group is one of the most studied taxa groups in stream ecology, as they have a long-lived larval stage connected to water habitat and a shorter-lived adult stage connected to terrestrial habitat [16]. However, stream ecosystems also host a diverse fauna of other macroinvertebrates, including representatives of taxonomic groups (e.g., Gastropoda, Crustacea, and Oligochaeta), which contribute to the overall ecological functioning of these environments [8].

The prevalence of feeding groups varies seasonally and reflects the ecological dynamics of these insect assemblages. The dominance of a particular feeding group (e.g., scrappers and collector–gatherers) probably benefits from increased primary production and the availability of organic matter, while shredders dominate when leaf litter input is higher [17]. These functional traits are essential for maintaining the structure and stability of aquatic insect communities, as they influence energy flow and nutrient cycling in stream ecosystems [18,19]. Understanding these seasonal shifts in the dominance of feeding groups provides valuable insights into the ecological functioning of mountain streams and their response to environmental changes [20].

Seasonal fluctuations in environmental variables, including water temperature, flow regime, and consequently, substrate type, are drivers of aquatic insect assemblages and functional traits in mountain streams. The flow regime depends on snowmelt in spring or precipitation in autumn [21]. A higher water level means a higher discharge, greater erosion of the streambeds, and differences in insect assemblages [22].

Changes in benthic macroinvertebrate communities can indicate changes in a stream’s biological integrity [23]; not only that, the diversity and abundance of target insect orders can provide comprehension of the overall functioning of mountain freshwater habitats [24].

In addition, changes in the functional structure of the community also provide important food information, along with a combination of environmental properties and behavioral mechanisms. Together with monitoring biodiversity, a trait-based approach is used to enhance understanding of stream ecology in terms of stream health and improve management and conservation practices [25,26].

In view of the above, three main objectives were set in this study: (1) to investigate the structure of aquatic insect assemblages, focusing on Ephemeroptera, Plecoptera, Trichoptera, Coleoptera, and Odonata, by assessing diversity and abundance in three mountain streams using taxonomic and trait-based approaches (through functional feeding groups); (2) to examine differences in the abundance and diversity of aquatic insect assemblages in relation to the effects of seasonal variability in environmental conditions; (3) to analyze the relationship between aquatic insect assemblages and site-specific hydrological and morphological conditions, revealing how these factors affected insects species diversity and distribution in mountain stream ecosystems.

## 2. Materials and Methods

### 2.1. Study Area

The study was conducted on three mountains of the Pannonian Lowland Ecoregion [27]: Papuk and Psunj mountains, located in eastern Croatia and forming a part of the Slavonian mountains [28] and the Medvednica mountain, in northwestern Croatia near the capital city of Zagreb. The distance between Medvednica and Psunj is about 130 km, while Papuk is right next to Psunj. The mountains analyzed differ mainly in their geology.

Papuk mountain, with its highest point reaching 913 m a.s.l., is a part of the mountain range that is considered a unique feature of the Slavonian area, which is mainly characterized by alluvial plains with elevations of about 100 m a.s.l. [28]. The remarkable ecological and geological richness of the Papuk mountain, which was declared a UNESCO World Heritage Site, led to its designation as a Nature Park in 1999 (NN 45/1999). With a protected area of 336 km^2^ (Papuk and a part of Krndija), it became the first Croatian Geopark [29]. From a geological point of view, there are various schists and granitoid rocks, which are an essential feature of this region [28,30].

Psunj mountain lies south-west of Papuk; however, it is not included within a protected area. Brezovo Polje (984 m a.s.l.) is the highest peak in the Slavonian mountains, its geology is similar to that of Papuk, composed of schists and granitoid rocks. This mountain range is important because it separates the watersheds of rivers Drava in the north, and Sava in the south [30]. The Papuk and Psunj mountains are remnants of the Paratethys Sea, which formed 16 million years ago and constituted the islands of the Slavonian archipelago [28,30].

**Figure 1:**
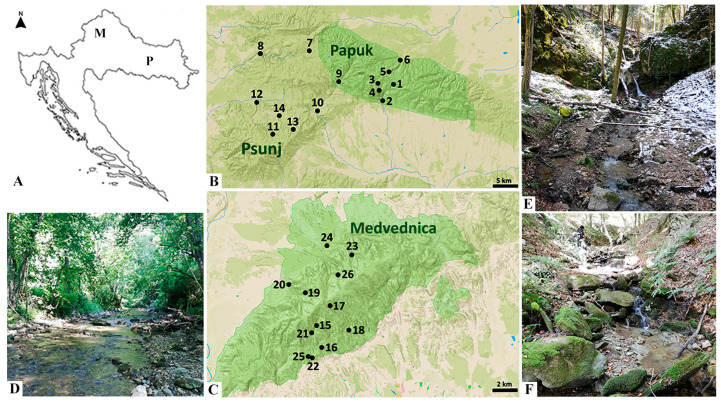
Study area. (**A**). Studied mountains in the Pannonian Lowland of Croatia: M-Medvednica, P-Papuk and Psunj. (**B**). Papuk and Psunj sampling sites: 1-Dubočanka HA, 2-Dubočanka LA, 3-Veličanka HA, 4-Veličanka LA, 5-Kovačica HA, 6-Kovačica LA, 7-Bijela HA, 8-Bijela LA, 9-Brzaja HA, 10-Brzaja LA, 11-Sivornica HA, 12-Sivornica LA, 13-Cikotska HA, 14-Cikotska LA. (**C**). Medvednica mountain sampling sites: 15-Kraljevec HA, 16-Kraljevec LA, 17-Bliznec HA, 18-Bliznec LA, 19-Bistra HA, 20-Bistra LA, 21-Mali Potok HA, 22-Mali Potok LA, 23-Rakova No-ga HA, 24-Vidak LA, 25-Veliki Potok LA, 26-Bistra 2 HA. (**D**). Sivornica LA in summer. (**E**). Bistra HA in spring. (**F**). Veličanka HA in autumn. In (**B**,**C**). The green area indicates a protected area of Nature Park or Geopark on both mountains. In the sampling site names, HA indicates site location at the higher altitude and LA at the lower altitude of the studied stream.

Medvednica was declared a Nature Park in 1981 (NN 24/1981) and covers an area of 179 km^2^, with Sljeme being the highest point in the area (1035 m a.s.l.). The geology of Medvednica is diverse and is known as a remnant of the Dinarides karst, so the geological base is dominated by flysch and metamorphic rocks layered on greenschist. It is also characterized by the presence of limestone rocks [31] (Figure 1).

### 2.2. Aquatic Insect Assemblage Analysis

The sampling of aquatic insects was carried out in the summer of 2020, and in the spring and autumn of 2021 at streams situated in three selected mountain regions in Croatia. The samples were taken at two different elevations, referred to as lower and higher altitude sites (Figure 1). Higher altitude sites were sampled at an elevation of more than 500 m a.s.l., whilst lower altitude sites were sampled at an elevation of less than 500 m a.s.l. A total of 26 sites on 14 streams were covered (Table 1) during three different seasons. Twelve sites were located in the Medvednica mountain region. The streams studied in the Medvednica area are as follows: the Bistra, Kraljevec, Bliznec, and Mali Potok streams (each stream was sampled at two elevations), and Vidak, Rakova Noga, and Veliki Potok streams (sampled only at a single elevation: Vidak and Veliki Potok LA, Rakova Noga HA). An additional third site at the Bistra stream was sampled only once during the summer season because sampling on other occasions was not possible due to closed access routes and mountain terrain that were too steep and dangerous. A total of 14 sites were investigated in the Slavonian mountains. In the area of the Papuk, the streams Veličanka, Dubočanka, Kovačica, and Bijela (each sampled at two elevations) as well as the upper stretches of the Brzaja stream, were analyzed. On Psunj, the samples of aquatic insects were collected from Sivornica and Cikotska (both streams were sampled at two elevations), and the Brzaja stream lower elevation site. All selected streams were anthropogenically undisturbed and hydrologically unaffected, and thus can serve as reference sites for further ecological studies [32].

The sampling was carried out according to the adapted AQEM protocol [33] using a benthos hand net (25 × 25 cm, mesh size 500 μm). The type of microhabitat substrates was defined according to Hering et al. [34]. The presence of different habitat types was assessed and three dominant microhabitats were selected. Each microhabitat, represented by three samples, was analyzed separately. In total, we had nine samples from each site. At the Mali Potok HA, samples of only two substrate types were collected as the third microhabitat type was not present.

All samples were preserved in a 96% ethanol solution. After fieldwork, from the macroinvertebrate samples, the specimens of Ephemeroptera, Plecoptera, Trichoptera, Odonata (larvae and nymphae), and Coleoptera (larvae and adults) were isolated and identified using stereomicroscope SMZ-171 (Motic, Hong Kong) and microscope BA310 (Motic, Hong Kong) to the lowest possible taxonomic level. The following identification keys were used [35,36,37,38,39]. Coleoptera was identified to the genus level, with the exception of one species, *Pomatinus substriatus* (Müller, 1806). During the identification process, the taxa were also categorized into functional feeding groups based on the classification according to Moog [40] and Merritt et al. [41]. Later, both larval and nymph stages are referred to as larvae.

### 2.3. Environmental Conditions Analysis

Basic physical and chemical water parameters such as water temperature, oxygen saturation, dissolved oxygen concentration, water conductivity, and pH value were measured in situ, using the probes, model HQ4300 (Hach, Germany). The water hardness was determined using 0.1 M HCl [42]. Also, we assessed the shading percentage over the sampled stream. The Croatian Meteorological and Hydrological Service provided data on monthly precipitation (rain or snow) from the measuring stations nearest to the sampled stream. Of the hydrological and morphological parameters, we measured sampling depth and water velocity for each of the nine samples, together with the width of the streambed and estimation of substrate type composition. The composition of the substrate type referred to the following: macrolithal (20 cm to 40 cm); mesolithal (6 cm to 20 cm); microlithal (2 cm to 6 cm); akal (0.2 to 2 cm); psammal (6 µm to 2 mm); xylal (dead wood, branches, roots); CPOM (Coarse Particulate Organic Matter).

### 2.4. Data Analysis

The data were analyzed using Primer 6 and CANOCO 4.5 software [43,44]. Prior to analysis, both matrices, the insect abundance, and the environmental variables, were log-transformed, and only the environmental variables were normalized. To form Resemblance matrices, we applied Bray–Curtis similarity to the taxa abundance data and Euclidean distance to the environmental data. Bray–Curtis accounts for species abundance, avoids artificial similarity, and highlights community differences. Euclidean distance measures straight distance and preserves absolute differences for clear environmental gradients [43]. To find the best subset of environmental variables as well as to explain the patterns observed in the aquatic insect assemblages, we applied BEST (Bio-Environmental Similarity Tool) analysis with Spearman’s rank correlations [45]. In addition, Shannon–Wiener (H’) [46] and Simpson’s (D) [47] diversity indices were calculated for each sampled stream using Primer 6 software.

Redundancy analysis (RDA) and distance-based redundancy analysis (dbRDA) were performed to identify the environmental variables explaining the variation in abundance of taxa and abundance related to the identified functional feeding groups in shaping the stream assemblages [43]. In addition, principal component analysis (PCA) was performed for all environmental variables analyzed expressing the main gradients of environmental conditions among the samples. The Monte Carlo permutation test with 999 permutations was used under the full permutation model to test the statistical significance of each environmental variable for all identified taxa [48].

In order to assess the relative contribution of each species to the average dissimilarity in species composition between groups of different mountains and different seasons as well, we performed a Similarity Percentage Partition (SIMPER). This method assesses the contributions of individual taxa to the differences between groups (seasons as temporal and mountains as spatial) based on Bray–Curtis dissimilarity [43,49]. To validate spatial and temporal gradients in assemblages, we used a Permutational Analysis of Multivariate Dispersions (PERMDISP) [48]. A Pairwise comparison with mean distance was calculated from the deviations from the centroid. The Monte Carlo permutation test with 999 permutations, under the full permutation model, was used to test the statistical significance [48]. In our study, groups refer to temporal (seasonal) and spatial (sampled mountains). The Bray–Curtis dissimilarity matrix and the log-transformation of abundance data were performed prior to the analysis. The level of significance was considered at *p* < 0.05.

## 3. Results

### 3.1. Aquatic Insect Assemblages

The macroinvertebrate analysis of 675 samples revealed a diverse aquatic insect community composition and structure, with a total of 130 taxa collected (across three mountains during three seasons); only one sample did not contain investigated aquatic insects. The highest number of taxa was recorded on Papuk (*n* = 113), followed by Medvednica (*n* = 110), and Psunj (*n* = 94). Trichoptera were the most diverse group, followed by Ephemeroptera, Coleoptera, Plecoptera, and Odonata, the latter representing the least diverse group. We identified 36 Ephemeroptera taxa (22 species), 15 Plecoptera (6 species), 59 Trichoptera (27 species), 6 Odonata (4 species), and 19 Coleoptera (one species).

The most abundant group on all three mountains was Ephemeroptera larvae. The most abundant taxa in the overall study in the streams of the Papuk mountain were as follows: *Leuctra* sp., *Baetis rhodani* (Pictet, 1843), *Baetis* sp., *Limnius* sp., *Elmis* sp., and the juvenile larvae from Heptageniidae and Hydropsychidae families, accounting for slightly less than 50% of all identified taxa. At Medvednica, Ephemeroptera larvae dominated, followed by Plecoptera and Trichoptera. *Protonemura montana* Kimmins, 1941 and *Leuctra* sp. were the most abundant, together accounting for slightly more than 25% of the community. The most dominant taxa in the streams on Psunj were: *Glossosoma conformis*/*boltoni* and *Leuctra* sp., which together comprised almost 20% of the total insect fauna.

### 3.2. Papuk Mountain Streams

On Papuk, Ephemeroptera larvae were the most abundant, with 35.13%, followed by Trichoptera (26.40%), Coleoptera (24.87%), Plecoptera (12.79%), and Odonata (0.80%). The most abundant Ephemeroptera species was *B. rhodani* (15.68%) while juvenile Heptageniidae larvae accounted for 20.28%. The most abundant Trichoptera taxa was *G. conformis*/*boltoni* (13.84%), with juvenile Hydropsychidae larvae (22.64%). *Elmis* sp (31.60%) and *Limnius* sp. (28.90%) were the most abundant Coleoptera taxa. *Leuctra* sp. (80.63%) was the most abundant Plecoptera taxon. Odonata was represented by six taxa: *Cordulegaster heros* Theischinger, 1979 (72.88%) and *Onychogomphus forcipatus* (Linnaeus, 1758) (22.71%) were the most dominant.

Ephemeroptera was the dominant group during all sampling seasons, and the least abundant individuals were Odonata. Plecoptera was more abundant in spring, while Trichoptera was more abundant during the summer and autumn sampling periods. The dominant Ephemeroptera species were *B. rhodani* and *Habrophlebia lauta* Eaton, 1884 in the summer, *B. rhodani* and *Baetis melanonyx* (Pictet, 1843), together with *Rhithrogena* gr. *semicolorata* (Curtis, 1834) in the spring period. And in the autumn, *R*. gr. *semicolorata*, *Habroleptoides confusa* Sartori and Jacob, 1986, and *B. rhodani* were the most abundant species.

### 3.3. Psunj Mountain Streams

Similar to Papuk, Ephemeroptera larvae were the dominant aquatic insect group in the mountain streams of Psunj with 35.56%, followed by Trichoptera (28.61%), Coleoptera (22.51%), Plecoptera (12.94%), and Odonata (0.38%), respectively. *Hydrocyphon* sp. (26.47%) and *Limnius* sp. (23.40%) were the dominant Coleoptera taxa. *B. rhodani* (15.59%) and *Baetis* sp. (14.88%) were the most numerous Ephemeroptera species, followed by the Heptageniidae family (14.46%) and *R*. gr. *semicolorata* (12%). The dominant Odonata species was *O. forcipatus* (79.66%). The representative of the Plecoptera was *Leuctra* sp. with 73.49%. As in Papuk, *G. conformis*/*boltoni* was the most dominant Trichoptera species, with 38.20% of all identified trichopterans, followed by the juvenile larvae of Sericostomatidae family (with *Sericostoma personatum*/*flavicorne*), which together accounted for 15.22%.

### 3.4. Medvednica Mountain Streams

The most frequently occurred aquatic insects in the mountain streams of the Medvednica were Ephemeroptera (42%), Plecoptera (31.74%), Trichoptera (15.34%), Coleoptera (10.77%), and Odonata (0.15%). *Baetis* species were the most abundant taxa (22.17%) with *B. rhodani* (14.64%) and Heptageniidae juvenile larvae (19.28%). The most abundant Coleoptera taxa were as follows: *Hydraena* sp. (23.35%), *Hydrocyphon* sp. (23.13%), and *Limnius* sp. (21.40%). Of the four Odonata taxa identified, *C. heros* dominated (84.10%), while *P. montana* (48.12%) and *Leuctra* sp. (34.53%) were the most abundant Plecoptera taxa. The most abundant Trichoptera taxa were Hydropsychidae juvenile larvae and Hydropsyche spp., which together accounted for 19.90%, *G. conformis*/*boltoni* (12.02%), and the species from the family Goeridae (11.19%).

### 3.5. Spatial and Seasonal Diversity

The Shannon–Wiener (H’) and Simpson’s (D) indices are shown in Table 2. The site with the highest aquatic insect diversity was the Dubočanka HA site in the autumn sampling period (Papuk). The lowest diversity was recorded at the Medvednica mountain stream site Bliznec HA in the spring sampling period.

The results of the SIMPER analysis showed a relative dissimilarity among stream communities in different seasons. The average similarity between samples within the summer sampling group was 35.35%. The species that contributed the most to the group similarity were the species from the *Baetis* genus, with the highest contribution of 14.21%, followed by *Leuctra* sp. with 13.42%. The average abundance of *Baetis* sp. was 1.77, indicating that it is a relatively common taxon in the summer samples. In spring, the average similarity was 31.45%, with the most common species being *P. montana* (average abundance: 1.54, 15.72%). In autumn, the average similarity was 34.75%, with *Leuctra* sp. being the most abundant taxon with an average abundance of 2.1 and 15.78%. The average dissimilarity between sampling periods was highest between spring and autumn (75.98%). SIMPER analysis revealed that species dissimilarity between Medvednica and Papuk streams was higher (72.98%) than the dissimilarity between the communities on Papuk and Psunj (66.80%).

### 3.6. Functional Feeding Groups

**Figure 2:**
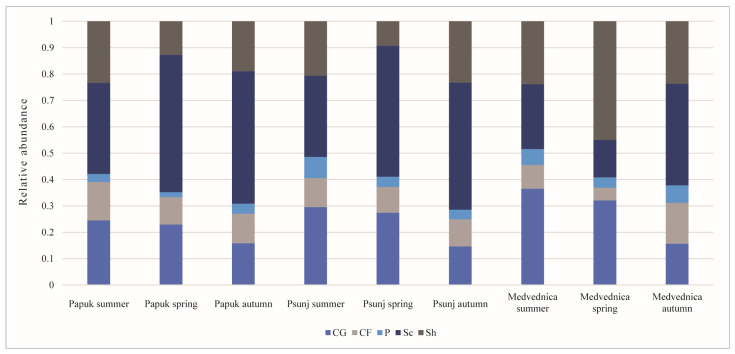
The composition of the functional feeding groups on three mountains in three seasons was presented in the relative number of individuals. Abbreviations: CG: collector–gatherers; CF: collector–filterers; P: predators; Sc: scrapers; Sh: shredders.

**Figure 3:**
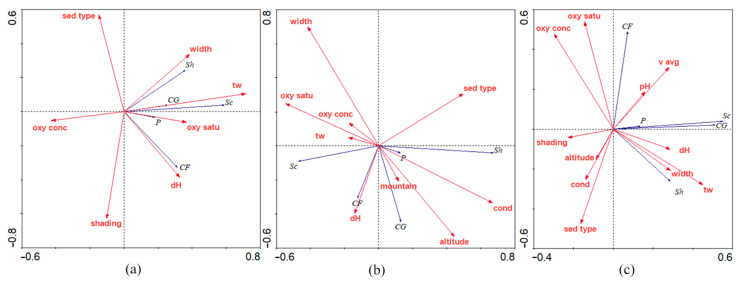
Redundancy analysis plot of functional feeding groups abundance corresponding to statistically significant environmental variables for each sampling season: (**a**) in summer, (**b**) in spring, and (**c**) in the autumn sampling period. Abbreviations: sed type—sediment type; width—streambed width (m); tw—water temperature (°C); oxy satu—dissolved oxygen saturation (%); dH—water hardness; shading—level of shading over the stream (%); oxy conc—oxygen concentration (mg/L); cond—conductivity (µS/cm); v avg—average water velocity (m/s).

The functional feeding group categories (FFG) in this study were as follows: collector–gatherers (CG), collector–filterers (CF), scrapers (Sc), shredders (Sh), and predators (P). Figure 2 shows the composition of the different functional feeding groups in the different seasons in the streams of three mountains. In the summer season, the dominant groups in the streams of all three mountains were collectors–gatherers and scrapers. In the spring season, scrapers dominated in the streams on Papuk and Psunj, but shredders were the dominant FFG in the streams of Medvednica. In the autumn period, the scrapers dominated again. The redundancy analysis showed a different correspondence of FFGs in the different seasons (Figure 3). Water temperature, streambed width, oxygen saturation, and water hardness were the most influential parameters in summer; in spring, FFGs are related to water hardness, altitude, and conductivity; and in autumn, the most correlated variables were water temperature, streambed width, and water hardness.

### 3.7. Environmental Conditions Affecting Aquatic Insect Assemblages Related to Seasonality

Eight substrate types were distributed at the sampling sites on all three mountains. If separation was not possible, three additional mixtures of two types were sampled. On the Papuk mesolithal (29.63%) and macrolithal (23.46%) substrate types dominated. On the Medvednica mesolithal (33.30%) dominated, closely followed by microlithal (23.23%), then macrolithal (12.12%), and xylal with CPOM (Coarse Particulate Organic Matter) (12.12%), whilst on the Psunj macrolithal and mesolithal were the most common, each totaled 33.30%.

Figure S1 shows the rainfall amounts during the study period. There was no unusual precipitation during the study or shortly before sampling. Summer was drier than autumn and spring. Precipitation was slightly higher on Medvednica than on Papuk and Psunj. Snow was only present during the winter months and early spring, and it could be related to the amount of water in the streams and affected hydrological conditions. The relative shading of the streams studied varied by season, and ranged from 26% to 58% in spring and 80% to 96% in summer and autumn.

**Figure 4:**
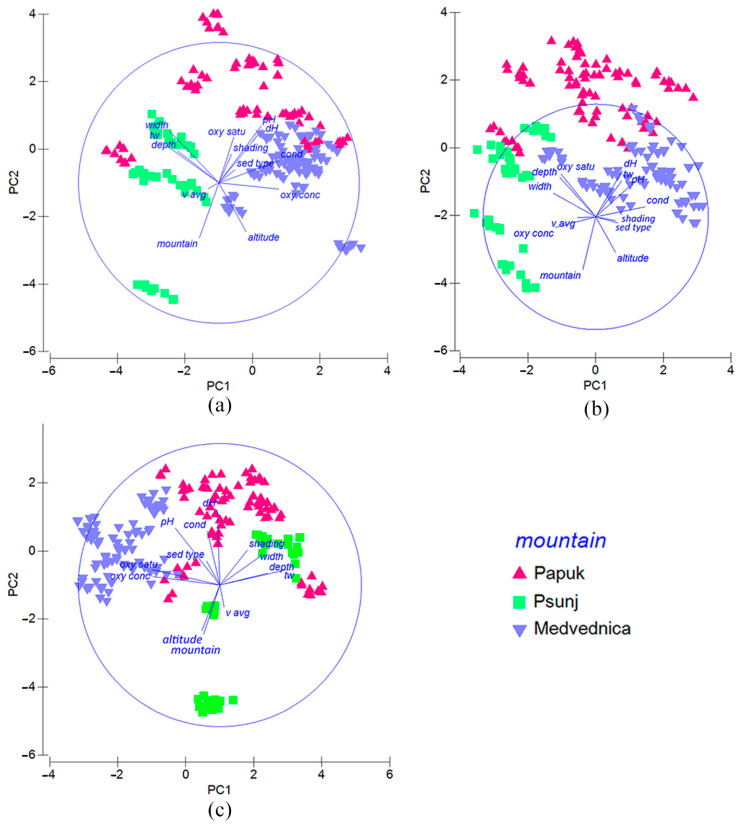
Principal component analysis (PCA) plot of the environmental, hydrological, and morphological variables analyzed at sampling sites in streams on three mountains. Different colors represent different groups of samples located in the mountains: (**a**) in summer, (**b**) in spring, and (**c**) in autumn season. Abbreviations as in Figure 3.

During the summer and spring sampling periods, all environmental variables were statistically significant (permutation test, 999 permutations, *p* < 0.05), with the exception of average water velocity in both periods and pH in spring. In autumn, all measured variables had a significant influence on aquatic insect assemblages. The composition of insect communities varied across different seasons due to various factors (Figure 4). In summer (Figure 4a), water temperature was the most important environmental variable (F value: 17.046, *p* < 0.001). In spring (Figure 4b), conductivity was the most influential variable (F value: 17.265, *p* < 0.001). Water temperature (F value: 8.833, *p* < 0.001) and pH (F value: 7.616, *p* < 0.001) had the greatest influence on the community structure in autumn (Figure 4c).

### 3.8. Environmental Parameters Influencing Aquatic Insect Assemblages as a Function of Spatial Factor

**Figure 5:**
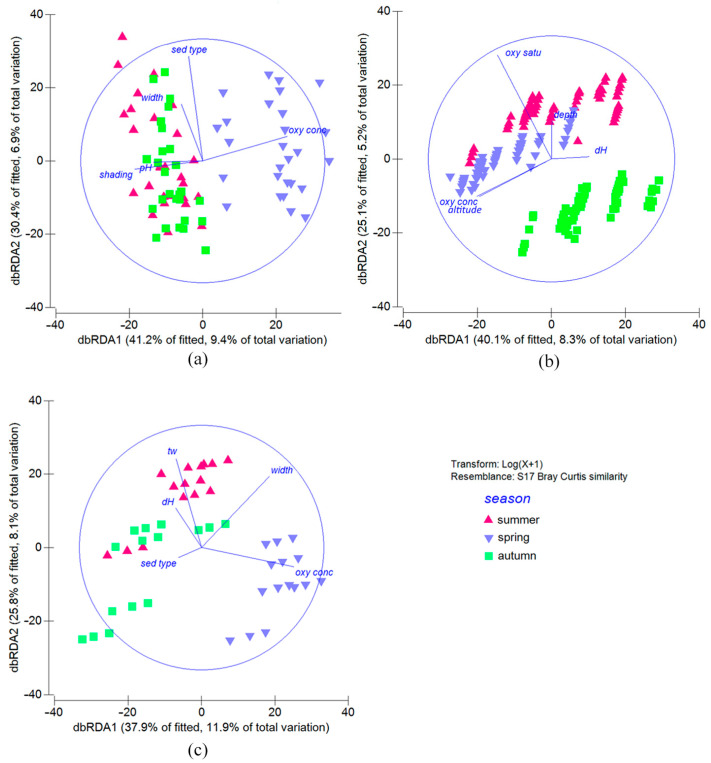
Distance-based redundancy analysis ordination of aquatic insect abundance data showing the relationships between samples and the best correlated environmental, hydrological, and morphological variables affecting the assemblages in different seasons (different symbols and colors correspond to different seasons): (**a**) abundance of studied streams on Papuk mountain, (**b**) on Medvednica mountain, (**c**) on Psunj mountain. Abbreviations as in Figure 3.

As the results of the correlation between environmental and taxa datasets, we found a weak positive correlation in the Papuk and the Medvednica datasets (0.385 and 0.311, respectively). However, the correlation of 0.497 for the aquatic insect assemblages of the Psunj showed that the combination of environmental variables analyzed explains a significant portion of the variability in species composition. The aquatic insect assemblages of the Papuk streams were correlated with shading, pH, oxygen concentration, sediment type, and streambed width the most. The first two axes could explain 71.60% of the taxa analyzed (Figure 5a). Only in Medvednica, the elevation correlated with the structure of aquatic insect assemblage (together with oxygen content, water hardness, and sampling depth). These variables can explain 65.20% of the aquatic insect assemblages (Figure 5b). The environmental and morphological variables with the highest correlations to the aquatic insect composition of the Psunj were as follows: water temperature, oxygen concentration, water hardness, sediment type, and streambed width. The dbRDA plot showed that the first two axes could explain 63.70% of the insects’ assemblages (Figure 5c).

Considering seasonality, a weak correlation was found in all the seasons examined (summer: 0.311; spring: 0.366; autumn: 0.279, respectively). In spring, we found a higher correlation with environmental variables: water temperature, oxygen saturation and sediment type, elevation, and sampling depth. The variables responsible for community assemblages during all seasons were sediment type and sampling depth.

The results of the PERMDISP analysis showed differences in multivariate dispersion among community groups, between seasons, and between mountain assemblages as well. The results are presented in Table 3, showing significant differences in multivariate dispersion between the three mountain groups overall (F-value: 3.4111, *p* = 0.044). The Papuk aquatic insect assemblages are significantly different from the aquatic insect assemblages of the Medvednica (t-value: 2.5926; *p* = 0.014), whilst the aquatic insect assemblages of the Psunj were not significantly different from either the Papuk or the Medvednica.

The PERMDISP analysis for each sampled season showed significant differences in the dispersion between summer, spring, and autumn (F-value: 16.974, *p* = 0.001). The composition of the aquatic insect assemblages in spring is statistically significantly different from the sampling in summer and autumn, whilst there were no statistically significant differences in the dispersion of the species composition between the sampling campaigns in the summer and autumn periods. The mean values for each season are shown in Table 4, suggesting that the spring sampling campaign had the highest mean value (51.962), possibly reflecting higher diversity in the data collected during this season.

## 4. Discussion

This study examines the composition and structure of benthic macroinvertebrate assemblages in mountain streams of the Pannonian Lowland Ecoregion in Croatia. The focus is on the seasonal variation in abundance and diversity of the key aquatic insect orders and their relationships to environmental variables, which contributes to our understanding of biodiversity in mountain streams and the role of aquatic insects in these streams. This can improve conservation strategies for these sensitive habitats. Mountain streams are in general, lotic ecosystems at medium (400 to 800 m a.s.l.) to high altitudes (1600 to 2500 m a.s.l.), with a continuous flow and significant seasonal fluctuations due to the main water source, which is either snowmelt or precipitation [50,51]. Although the streams in this study are below these altitudes (min. 241 to max 819 m a.s.l.), they still harbor dominant cold water stenotherms such as Ephemeroptera, Plecoptera, and Trichoptera, probably due to the diversity of microhabitats [52]. In general, the dominant substrate in these stream-types consists of various-sized stones, cobbles, and gravel [53], which supports the study objectives. The dominant substrate type differs according to altitude, and mountain range, and mainly influences the community structure.

### 4.1. Contribution to Species Diversity

Ephemeroptera species were the most abundant aquatic insect group, with species from Baetidae and Heptageniidae families as dominant taxa. During their surveys on Papuk, Vilenica et al. [54], recorded *Ephemera danica* Müller, 1764 and *Baetis rhodani* as the most frequent species in streams. Both species were also found during our research. The high abundance and frequency of Ephemeroptera reflect the presence of their favorable microhabitat and substrate characteristics, and the fact that the studied streams are not yet exposed to anthropogenic pressure [54,55,56]. According to the study by Previšić et al. [57] and our study, the streams on Papuk are characterized by low water temperature and high velocity, which correspond to the mountain stream type as well as the presence of rheophilic Ephemeroptera species, such as *Baetis melanonyx*, *B. rhodani* and *Rhithrogena* gr. *semicolorata*, which occurred in large numbers. *Rhithrogena* species are indicator species for water quality as they live in well-oxygenated, fast-flowing streams, and have a low tolerance to pollution [18,56].

Even though they were not present in large numbers, it is important to point out the Odonata species found in this study, *Cordulegaster heros*, which was found in all three mountains’ streams, accompanied by plecopteran *Taeniopteryx hubaulti* Aubert, 1946 on the Psunj mountain. These two species are known to inhabit clean, well-oxygenated streams and rivers, and both are listed as endangered (EN) species according to the IUCN Red List [58,59]. The factors threatening these species include habitat degradation, pollution, and climate change, which could have a negative impact on the quality of these species’ freshwater habitats. This information on endangered species benefits our aim of investigating potential reference sites with zero anthropogenic influence. In addition, *C. heros* is a species endemic to south-east Europe [60,61,62]. In general, Odonata are an essential link in the food webs, since larvae and adults are among the most effective predators in freshwater ecosystems [63,64]. The low diversity of Odonata in this study is most likely due to the habitat type sampled and the lack of different types of aquatic vegetation [35,65,66,67]. During these surveys, we found *C. heros* on all investigated mountains’ streams, and the species *Cordulegarster bidentata* was found on Papuk and Medvednica mountains, but not on Psunj, even though Papuk and Psunj have similar assemblage composition. The most abundant Odonata species in all studied streams were *C. heros* and *Onychogomphus forcipatus*. According to a study by Vilenica et al. [68], *Calopteryx virgo* (Linnaeus, 1758) was the most widespread species in the area of the Papuk Nature Park, together with *O. forcipatus* and *C. bidentata*.

The proof that the streams at Papuk mountain are of particular importance and that the area is not yet sufficiently explored is recently described new plecopteran species, *Leuctra papukensis* Reding, Vinçon and Graf, 2023. This species is very sensitive to pollution and prefers to inhabit only clean streams. *L. papukensis* is currently only known from the Jankovac stream, which flows into the mouth of the Kovačica [69].

Diversity indices revealed that both the Shannon–Wiener and the Simpson index in Medvednica had lower values (Bliznec HA site in spring with SW 1.435) than in Papuk (Dubočanka HA site in autumn with SW 3.103) and Psunj at all sampling sites. Marion et al. [70] found that wilderness areas are degraded by recreational users. This finding might be related to the fact that Medvednica is near the most populous city in Croatia, the City of Zagreb and that it is more visited mountain than Papuk and Psunj. According to the Medvednica visitor website, the total number of visitors is around one million people per year. The Medvednica area is a popular destination for the people of Zagreb to relax and socialize throughout the year.

### 4.2. Spatial and Seasonal Variability in Aquatic Insect Assemblages

The aquatic insect assemblages showed greater similarity between Papuk and Psunj mountain streams than those in Medvednica. This is probably a consequence of two important factors, better geographical connectivity of the mountains and the differences between the prevailing substrate types. An example of spatial connectivity was the Brzaja stream, whose higher site on the Papuk was compared with its lower site on the Psunj. Considering all analyses, the aquatic insect assemblage composition of the Brzaja stream at higher elevations was more similar to the samples collected on Psunj mountain, than those on Papuk mountain (Figure 4). Considering the differences in the assemblage composition between the mountains in relation to the prevailing microhabitats, a similar pattern was found between Papuk and Psunj. Macrolithal combined with mesolithal substrate types dominated there, and in Medvednica streams organic substrate was dominant, i.e., xylal and CPOM. The heterogeneity of the habitat, primarily influenced by altitude, creates diverse microhabitats that result in insect biodiversity hotspots [8,9], ultimately contributing to the high diversity of all fauna in mountain streams and stream health [2,10]. Not only the taxonomic structure of the insect assemblages was influenced by the predominant substrate, but also the functional structure, since xylal and CPOM can be excellent direct food sources [71].

In spring, in the mountain streams of Medvednica, shredders were the dominant FFG compared to scrapers, which dominated the streams of Psunj and Papuk. We also recorded a higher similarity of assemblages in summer and autumn compared to assemblages in the spring period. Our results related to FFGs from three different mountain streams during three different seasons are consistent with several recent studies that showed that FFGs affiliation in benthic macroinvertebrates varied across seasons, indicating that most insects are opportunistic feeders [72]. We found a higher abundance of plecopteran species in streams on Medvednica, especially taxa such as *Leuctra* sp., *Nemoura* sp., and *Protonemoura montana*, which are benthic species belonging to the FFG shredders and correspond to the presence of CPOM [19] as one of the dominant substrates on Medvednica.

Most of the study sites are located in wooded areas and shading changes depending on the season, which affects the composition of aquatic insects in the stream itself. In summer and autumn, shading was higher than in spring, when there was no shading at all. That is why the distribution of FFGs was influenced similarly by other environmental variables, primarily water hardness and streambed width. In ecological research to date, the consideration of shading as a parameter influencing behavior and feeding preferences is of growing interest [73]. Shading plays an important role in predator–prey interactions, and light availability in streams can affect the abundance and diversity of periphyton (e.g., algae and microorganisms), which serve as food for many macroinvertebrates [74,75]. Consequently, shifts in light and shade can lead to changes in the feeding preferences and rates of stream organisms, which were also observed in our study.

### 4.3. Influence of Environmental Parameters

The aquatic insect assemblages of three studied mountains’ streams varied as a response to changes in environmental variables. As this was a comprehensive study analyzing the structure of aquatic insect populations in mountain streams, the results presented here show how stream ecosystems can respond to changing environmental factors. The results showed that seasonal fluctuations strongly influence aquatic insect assemblages in mountain streams and that the response of assemblages to environmental variables is influenced by morphology and hydrology rather than basic physical and chemical water quality parameters.

For the aquatic insect assemblages, we found the highest correlation in environmental variables, both explaining the shape and structure of the Psunj insect community. The moderate correlation between the measured environmental variables and assemblage structure on Papuk and Medvednica suggested that whilst these variables are much more important, and the results were statistically significant, other factors should also be considered. Similar results were found for the structure of dipteran assemblages in the streams of these three mountains. The dipteran assemblages of Medvednica and Papuk were more influenced by spatial variables than by the environmental variables analyzed [76,77].

Although elevation was a statistically significant parameter only for the aquatic insect assemblages on the Medvednica, we assumed that differences in elevation indirectly affect other parameters, such as substrate type and oxygen content [78]. These parameters sequentially affected the composition of aquatic insects in the streams of all three mountains. Additionally, our results suggest that seasonal variations in water temperature, average shading over the sampling site, and conductivity, affected the distribution of aquatic insects in all studied mountains’ streams. Together with elevation, precipitation patterns may differ, particularly in terms of snowfall. On Medvednica, more centimeters per square meter of snow and rain fell during the sampling period than on Papuk and Psunj, influencing the availability of habitats and the aquatic insect assemblages.

Understanding the relationships between precipitation and flow regimes in mountain streams is crucial for understanding the composition of aquatic insect assemblages. Fluctuations in precipitation change the flow patterns of streams. An increase in precipitation in autumn and spring can increase discharge rates and alter sediment transport and habitat viability for aquatic macroinvertebrates [79]. Our results suggest that this could also be one of the reasons why the aquatic insect assemblages differ between spring and autumn compared to summer, together with the results of PERMDISP, which showed a higher heterogeneity of assemblages during the spring period. This means that higher variability and diversity of macroinvertebrates were observed in spring. Similar results were also observed in the study Milošević et al. [80]. We assume that this is related to the emergence patterns of the insect species studied, which can occur to a greater extent from June to August [81], whilst no emergence had yet taken place in April during our spring sampling period.

These observed seasonal variations in assemblages highlight the potential impact of future climate change-driven shifts in precipitation and flow regimes on mountain streams. As the average air temperature rises, an increase in precipitation during the rainy season is observed, as well as more frequent and prolonged periods of drought in the summer months [82]. Generally, an increase in precipitation amount and intensity leads to erosion of mountain streambeds and changes in the substrate. This influences benthic communities since sediment type is one of the key structuring elements [83]. In addition, species disappear completely during dry periods when streams dry up. In this way, mountain streams at lower altitudes, which were the subject of our research, are more affected. Consequently, the trophic structure changes as well. More different-sized organic matter available in the aquatic system leads to more favorable conditions for filter feeders and shredders [84].

## 5. Conclusions

The present study showed that aquatic insect assemblages in mountain streams respond more strongly to morphological factors (e.g., substrate type, streambed width, and elevation) and less to the physical and chemical water variables (e.g., conductivity). While some environmental variables significantly affected assemblage structure, the unexplained variability suggests that further research is needed on other factors, such as exposure to the mountain, sedimentation, nutrient content, and stream sinuosity, or a better explanation of spatial dynamics using different models such as metacommunity approach, which links spatial proximity to community composition. These results provide valuable insights into the ecology of streams. They also emphasize the conservation importance of mountain streams as refugia for aquatic mountain insect species of the Pannonian Lowland Ecoregion in Croatia. Important findings of endangered insect species, such as *Cordulegaster heros* and *Taeniopteryx hubaulti*, further confirm these conclusions. The results of the current study contribute to the knowledge of biodiversity in mountain streams and to the understanding of the role of aquatic insects in streams, thus improving conservation strategies for these sensitive habitats that are threatened by different pressures worldwide, such as global climate change or different types of pollution.

## Figures and Tables

**Table 1:** Sampling sites on the analyzed mountains with geographical coordinates, elevation, and dominant substrates at the sampling sites (HA—higher altitude site; LA–lower altitude site). Grain size of inorganic substrate: macrolithal (20–40 cm); mesolithal (6–20 cm); microlithal (2–6 cm); akal (0.2–2 cm); psammal (6 µm–2 mm); xylal—dead wood, branches, roots; CPOM—Coarse Particulate Organic Matter.

Mountain	Site Number	Sampling Sites	Latitude	Longitude	Elevation (m)	Substrate Type Sampled		
Papuk	1	Dubočanka HA	45°29′53.0402″ N	17°41′06.3962″ E	510	macrolithal	mesolithal	akal
	2	Dubočanka LA	45°28′05.0584″ N	17°39′25.7028″ E	313	macrolithal	mesolithal	akal/ psammal
	3	Veličanka HA	45°29′58.9226″ N	17°38′39.9127″ E	540	mesolithal	microlithal	phytal (moss)
	4	Veličanka LA	45°29′12.0196″ N	17°38′51.6752″ E	350	macrolithal	mesolithal	akal
	5	Kovačica HA	45°31′16.2020″ N	17°40′25.8704″ E	587	mesolithal	microlithal	akal/ psammal
	6	Kovačica LA	45°32′34.6322″ N	17°42′12.7032″ E	216	macrolithal	mesolithal	akal/ psammal
	7	Bijela HA	45°33′37.5947″ N	17°27′40.2850″ E	567	macrolithal	mesolithal/ microlithal	psammal
	8	Bijela LA	45°33′19.6970″ N	17°19′52.9951″ E	273	mesolithal	microlithal	akal/ psammal
	9	Brzaja HA	45°30′08.4380″ N	17°32′23.6873″ E	326	macrolithal	mesolithal	psammal
Psunj	10	Brzaja LA	45°26′55.7606″ N	17°28′58.9086″ E	241	macrolithal	mesolithal	psammal
	11	Sivornica HA	45°24′17.3901″ N	17°21′50.3550″ E	765	macrolithal	mesolithal	microlithal
	12	Sivornica LA	45°27′52.4345″ N	17°19′18.4885″ E	334	macrolithal	mesolithal	microlithal
	13	Cikotska HA	45°24′50.71″ N	17°25′08.68″ E	725	macrolithal	mesolithal	akal
	14	Cikotska LA	45°26′24.2445″ N	17°22′53.2382″ E	366	macrolithal	mesolithal	microlithal
Medvednica	15	Kraljevec HA	45°52′54.9058″ N	15°56′33.7911″ E	589	mesolithal	microlithal	xylal/CPOM
	16	Kraljevec LA	45°51′56.4801″ N	15°56′53.9527″ E	384	mesolithal	microlithal	xylal/CPOM
	17	Bliznec HA	45°53′48.9760″ N	15°57′25.5210″ E	819	macrolithal	mesolithal	xylal/CPOM
	18	Bliznec LA	45°52′43.7938″ N	15°58′37.0039″ E	402	macrolithal	mesolithal	microlithal
	19	Bistra HA	45°54′22.3981″ N	15°55′49.4613″ E	519	macrolithal	mesolithal	akal/ psammal
	20	Bistra LA	45°54′45.7918″ N	15°54′45.2363″ E	320	macrolithal	mesolithal	akal/ psammal
	21	Mali Potok HA	45°52′35.6642″ N	15°56′13.4719″ E	593	mesolithal	xylal/CPOM	
	22	Mali Potok LA	45°51′28.5240″ N	15°56′09.8860″ E	310	mesolithal	microlithal	xylal/CPOM
	23	Rakova Noga HA	45°56′05.5498″ N	15°58′49.6007″ E	534	mesolithal	microlithal	xylal/CPOM
	24	Vidak LA	45°56′30.6217″ N	15°57′14.7400″ E	315	mesolithal	microlithal	xylal/CPOM
	25	Veliki Potok LA	45°51′29.7565″ N	15°56′05.7794″ E	301	mesolithal	microlithal	xylal
	26	Bistra 2 HA	45°55′12.1207″ N	15°57′56.1521″ E	694	macrolithal	mesolithal	microlithal

**Table 2:** Values of the diversity indices at sampling sites in different seasons. In bold are the maximum values of the calculated indices. *—No sampling was carried out.

Sampling Site	Shannon–Wiener (H’)			Simpson’s (D)		
	Summer	Spring	Autumn	Summer	Spring	Autumn
Dubočanka HA	2.526	2.632	**3.103**	0.851	0.892	**0.944**
Dubočanka LA	**2.988**	2.655	2.807	**0.933**	0.891	0.918
Veličanka HA	2.255	2.714	2.535	0.780	0.878	0.820
Veličanka LA	2.893	2.454	2.695	0.909	0.834	0.899
Kovačica HA	2.388	2.673	2.529	0.843	0.898	0.878
Kovačica LA	2.486	**2.870**	2.85	0.863	**0.916**	0.904
Bijela HA	2.848	2.788	2.536	0.919	0.914	0.869
Bijela LA	2.280	2.268	2.865	0.813	0.833	0.920
Brzaja HA	2.718	2.674	2.843	0.857	0.893	0.918
Brzaja LA	2.954	**2.967**	**2.942**	0.919	**0.931**	**0.924**
Sivornica HA	**2.956**	2.346	2.782	0.914	0.846	0.902
Sivornica LA	2.733	2.764	2.249	0.902	0.908	0.821
Cikotska HA	2.855	2.325	2.455	**0.926**	0.784	0.857
Cikotska LA	2.891	2.866	2.839	0.918	0.902	0.897
Kraljevec HA	2.663	1.931	2.761	0.898	0.728	0.902
Kraljevec LA	1.740	1.621	2.712	0.741	0.711	0.901
Bliznec HA	2.47	1.435	2.473	0.862	0.602	0.846
Bliznec LA	2.446	2.042	2.329	0.884	0.789	0.853
Bistra HA	2.617	2.023	2.801	0.901	0.793	0.911
Bistra LA	1.962	1.996	2.754	0.743	0.784	0.912
Mali Potok HA	2.106	1.978	2.532	0.824	0.790	0.897
Mali Potok LA	2.023	2.417	2.856	0.774	0.879	0.916
Rakova Noga HA	2.245	2.556	**2.986**	0.766	0.867	**0.934**
Vidak LA	1.954	2.570	2.587	0.719	0.874	0.869
Veliki Potok LA	**2.687**	**2.636**	2.286	**0.905**	**0.894**	0.819
Bistra 2 HA	2.030	*	*	0.822	*	*

**Table 3:** PERMDISP analysis of aquatic insect assemblages. t: deviation from centroid; in bold are statistically significant values based on the Monte Carlo Permutation Test, *p* < 0.05.

Sampling Campaign Groups	t	*p*-Value
Papuk, Medvednica	2.5926	**0.014**
Papuk, Psunj	0.7627	0.475
Medvednica, Psunj	1.3882	0.181
summer, spring	4.6651	**0.001**
summer, autumn	0.6674	0.553
spring, autumn	5.4371	**0.001**

**Table 4:** PERMDISP analysis of aquatic insect assemblages within a defined group. Average and SE (standard error) are the mean distances from group centroids.

Sampling Campaign	Average	SE
summer	47.920	0.62207
spring	51.962	0.60177
autumn	47.343	0.59951
Papuk	49.245	0.62672
Medvednica	51.269	0.48503
Psunj	50.028	0.78804

## Data Availability

Data are available upon request from the authors.

## Supplementary Materials

The following supporting information can be downloaded at https://www.mdpi.com/article/10.3390/insects16040344/s1. Figure S1. The amount of precipitation in the study area on three mountains from June 2020 to December 2021. The columns represent the amount of snow, and the lines represent the amount of rain from the nearest rain stations to the studied streams. The data was provided by the Croatian Meteorological and Hydrological Service. The values are expressed in millimeters of rain and snow per square meter.
