# Supplementary figures and images for: Seasonal Dynamics and Factors Shaping Aquatic Insect Assemblages in Mountain Streams of the Pannonian Lowland Ecoregion
Source: Insects. 2025 Mar 26;16(4):344. doi: 10.3390/insects16040344 (PMC12028206; doi:10.3390/insects16040344)

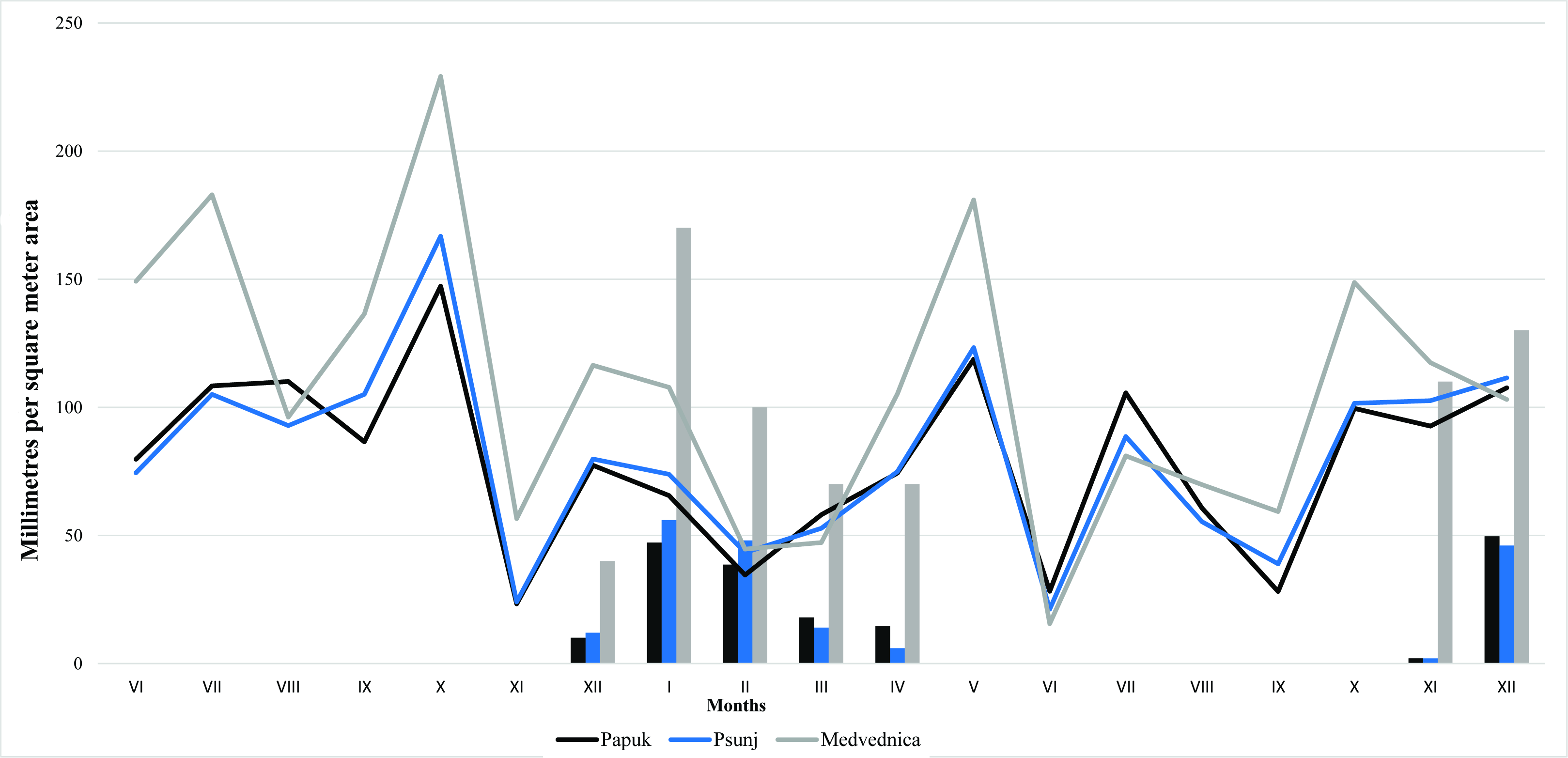

Supplement: Supplementary file 1 [file insects-16-00344-s001.zip › insects-3449369-supplementary.tif]
